# Thrombocytosis with acquired von Willebrand disease in an adolescent with sickle cell disease

**DOI:** 10.1002/ccr3.3556

**Published:** 2020-11-20

**Authors:** Marianne E. M. Yee, Glaivy Batsuli, Satheesh Chonat, Sunita Park

**Affiliations:** ^1^ Aflac Cancer and Blood Disorders Center Children's Healthcare of Atlanta Atlanta GA USA; ^2^ Department of Pediatrics Division of Hematology/Oncology Emory University School of Medicine Atlanta GA USA; ^3^ Department of Pathology and Laboratory Medicine Children's Healthcare of Atlanta Atlanta GA USA

**Keywords:** acquired Von Willebrand disease, priapism, sickle cell disease, thrombocytosis

## Abstract

Thrombocytosis is common in sickle cell disease and may contribute to vaso‐occlusion. Hydroxyurea treats extreme thrombocytosis. Acquired von Willebrand disease should be considered prior to aspirin therapy.

## Graphical abstract

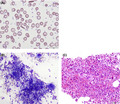

## 1 INTRODUCTION

Thrombocytosis in children is commonly a reactive condition secondary to inflammation, infection, iron deficiency anemia, or asplenia^1^, ^2^ and rarely requires intervention. Conversely, primary thrombocytosis is uncommon in pediatrics, but may represent an acquire myeloproliferative disorder or familial thrombocytosis,^3^ conditions that may be associated with thrombosis and bleeding.^4^

Thrombocytosis is common in children with sickle cell disease (SCD). Reactive thrombocytosis must be distinguished from myeloproliferative disorder or inherited thrombocytosis syndrome. We present an adolescent with hemoglobin SS and thrombocytosis associated with increased frequency of pain, priapism, and acquired von Willebrand disease.

## 2 CASE PRESENTATION

A 16‐year‐old boy with hemoglobin SS sickle cell disease (SCD) presented with vaso‐occlusive pain and extreme thrombocytosis (platelet count 2 428 000‐2 795 000/µL). History was significant for frequent episodes of pain and priapism and intermittent thrombocytosis over the 12 months prior to this presentation (range 228 000‐1 885 000/µL; mean 927 000/µL). Corporal irrigation with phenylephrine injection for priapism was required 4 times in the past year. There were no neurological disturbances or gastrointestinal symptoms. Physical examination and abdominal ultrasound showed no lymphoproliferation, hepatosplenomegaly, or thrombosis. Hydroxyurea therapy at 20 mg/kg had been started 18 months prior, and low‐dose aspirin therapy had been administered intermittently for 10 months. He had a lifetime transfusion history of 3 packed red blood cell (RBC) units with no transfusion in the past year.

**FIGURE 1:**
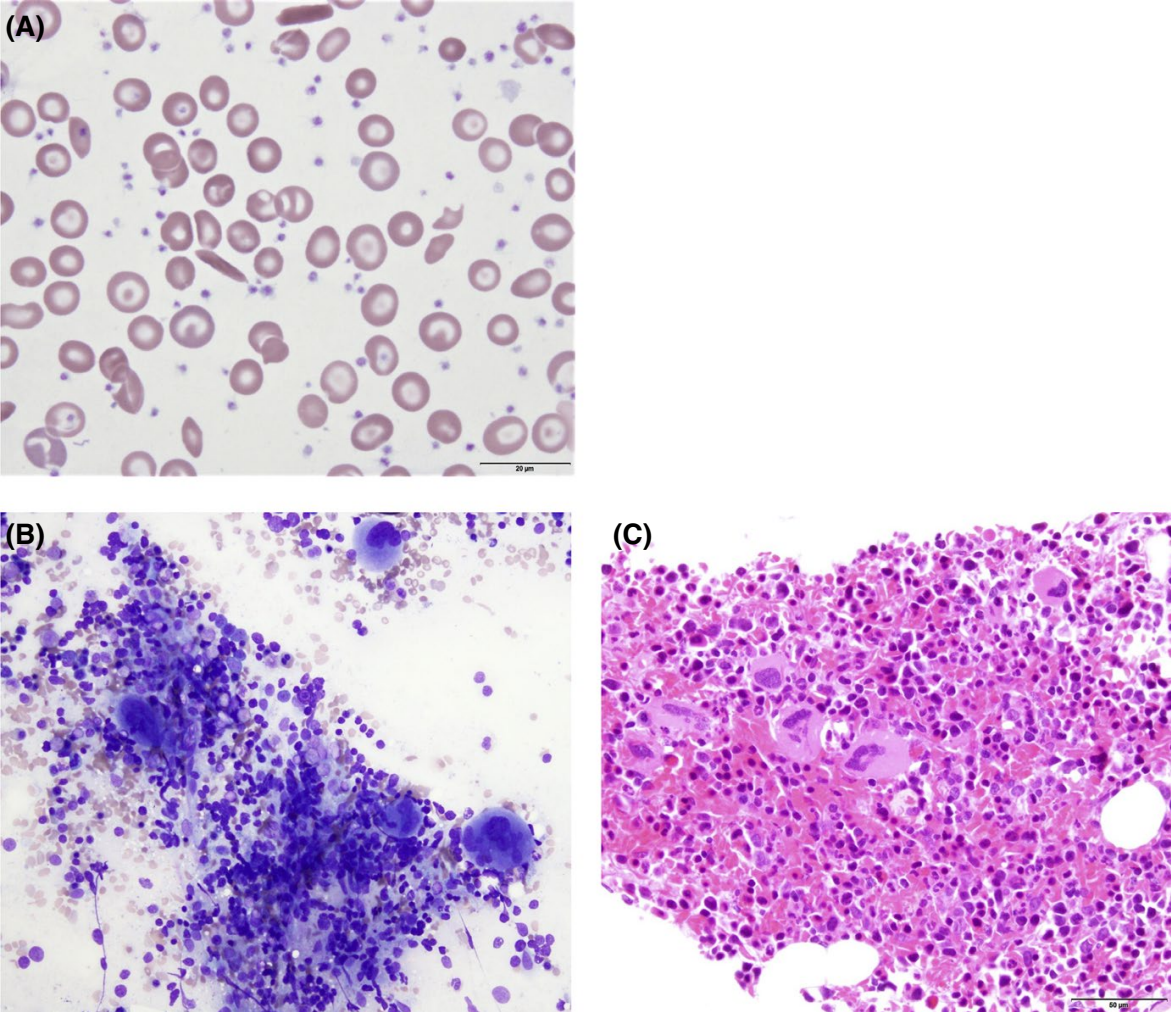
Histopathologic findings of sickle cell disease and reactive thrombocytosis. A, Peripheral blood smear showed sickle cells, target cells, hypochromic RBC, and increased platelets (Wright‐Giemsa, ×100). B, Bone marrow aspirate showed mild erythroid megaloblastic changes and increased megakaryocytes with hyperlobated forms (Wright‐Giemsa stain, ×40). C, Bone marrow core biopsy showed increased cellularity (Wright‐Giemsa stain, ×20)

White blood cell count was 10.15 × 10^3^/µL, hemoglobin 9.3 g/dL, mean corpuscular volume (MCV) 80.9 fL, and fetal hemoglobin 12.1%. Serum ferritin was 100 ng/mL, total iron binding capacity 290 µg/dL, serum iron 78 µg/dL, and iron saturation 27%. Peripheral blood smear (Figure 1, panel A) demonstrated sickle cells, target cells, a subset of hypochromic red blood cells (RBC), markedly increased platelets. Bone marrow aspirate smear (Figure 1, panel B) showed a decreased myeloid:erythroid ratio (<1:1) with normal myeloid maturation. Erythroid maturation showed mild megaloblastic changes. Megakaryocytes were present in increased number with occasional hyperlobated forms. Iron stain of the aspirate smear showed absent iron stores, and no ringed sideroblasts. Bone marrow core biopsy (Figure 1, panel C) showed increased cellularity of 90%, minimal reticulin fibrosis, and no evidence of dysplasia. Peripheral blood BCR‐ABL fluorescent in situ hybridization was negative.

Genetic sequencing with deletion and duplication analysis of the following genes associated with myeloproliferative disorders and thrombocytosis identified no variants: *JAK2*, *CALR*, *MPL*, *ABL1*, *BCR*, *AR*, *CBL*, *CEBPA*, *IDH1*, *IDH2*, *SF3B1*, *SRSF2*, *TET2*, *THPO*. BCR/ABL1 mRNA qualitative analysis was negative. Thrombopoietin (TPO) level was 8 pg/mL (reference range 7‐99 pg/mL).

Platelet function analysis performed using the PFA‐100 while on aspirin therapy demonstrated a collagen‐ADP closure time of 82 seconds, collagen‐EPI closure time of >300 seconds with platelet count 2 533 000/µL. Von Willebrand factor (VWF) testing showed low VWF ristocetin cofactor activity (VWF:RCoF) and decreased high molecular weight (MW) multimers (Table 1). For treatment of severe thrombocytosis, the hydroxyurea dose was increased to 30 mg/kg daily. Aspirin therapy was continued, and oral iron therapy was initiated. Repeat VWF testing occurred 9 days later, including VWF:Glycoprotein Ib (GPIb) measured by gain‐of‐function mutant GPIb binding assay (VWF:GPIbM). Platelet count decreased to normal; however, intermittent thrombocytosis >1 000 000/µL continued. Approximately 6 months later, VWF testing showed normal antigen and VWF:RCoF levels, normal VWF GPIbM activity, and normal multimer pattern. Priapism frequency and severity have decreased. He has had no evidence of bleeding on aspirin therapy.

**TABLE 1:** Acquired von Willebrand disease testing

	Initial	9 d later	24 wk later
Platelet count (per µL)	2 533 000	1 655 000	663 000
VWF antigen (%)	98%	70%	114%
VWF ristocetin cofactor (%)	36%	35%	70%
Factor VIII activity (%)	217%	154%	180%
High MW multimers (%)	9%	15%	18%
Intermediate MW multimers (%)	51%	52%	55%
Low MW multimers (%)	40%	33%	27%
VWF GPIbM activity (IU/dL) *Reference 60‐184 IU/dL*	NT	58	102%

Note

Changes in VW testing over time, as platelet count decreased with increased hydroxyurea therapy.

Abbreviation: NT, not tested.

## 3 DISCUSSION

Reactive thrombocytosis is a common finding in SCD and may contribute to the pathogenesis of vaso‐occlusion.^5^ Iron deficiency also results in thrombocytosis which improves with iron repletion.^1^, ^6^ In SCD, iron deficiency may be difficult to detect, as serum ferritin may be elevated due to inflammation. Diagnosis of iron deficiency may be further complicated if microcytosis is obscured by hydroxyurea therapy. The absence of bone marrow iron stores was suggestive of iron deficiency in this patient, although it is not always predictive of iron deficiency.^7^

In cases of extreme thrombocytosis, etiologies other than reactive thrombocytosis must be considered, including myeloproliferative neoplasms (MPNs) such as essential thrombocythemia (ET), polycythemia vera, and primary myelofibrosis (PMF) as well as inherited thrombocytosis syndromes. The specific genetic testing selected for this patient served to assess for such conditions. The *BCR/ABL* translocation was tested to identify chronic myelogenous leukemia (CML). Among non‐CML MPNs, the majority have the Janus kinase 2 gene (*JAK2*) V617F mutation, while *JAK2* wild‐type patients with ET and PMF commonly have mutations in exon 9 of the calreticulin gene *CALR*, and approximately 5% of adults carry *MPL* exon 10 mutations.^8^, ^9^ Given that pediatric ET is rare and heterogenous, seldom involving the classic 3 MPN genes described above, we additionally explored less common genes associated with ET including *IDH1, IDH2*,^10^ *TET2*, *SRSF2*,^11^ *SF3B1*,^12^ *CBL*, *CEBPA*,^13^ and *AR* encoding the androgen receptor. Mutations in the thrombopoietin gene (*THPO*) are associated with autosomal dominant familial ET.^14^ Serum TPO level is an important screening test for ET, particularly familial ET if markedly elevated above 1000 pg/mL.^3^ TPO in this case was low normal, which suggests that inflammation was an unlikely driver for thrombocytosis. Though the genetic testing performed is not exhaustive and does not exclude other germline or somatic mutations in unidentified genes, our testing did exclude some of the most common reported genetic alterations causing extreme thrombocytosis. Patients with ET are prone for vascular complications, and SCD adds additional risk for stroke and thrombosis. Our patient did not experience thrombosis during his period of platelet count >2 000 000/µL, which may be partly attributed to the use of hydroxyurea and low‐dose aspirin.

A consequence of severe thrombocytosis >1 000 000/µL may be acquired von Willebrand disease (VWD), as characterized by reduced VWF activity and loss of high MW VWF multimers, due to excessive binding of platelet GPIb receptors to circulating VWF.^15^, ^16^ The VWF:GPIbM assay measures the binding of VWF with platelet GPIbα, providing a sensitive, functional assay of VWF activity that correlates with the VWF:RCoF assay but is not subject to falsely low levels seen in common polymorphisms of the ristocetin binding regions of VWF, such as the D1472H polymorphism.^17^, ^18^ The VWF:GPIbM activity level in our patient during the time of extreme thrombocytosis remained above the level of probable bleeding risk identified by the National Heart Lung and Blood Institute Expert Panel report on VWD.^19^, ^20^ The use of aspirin as an antiplatelet agent in our patient was carefully considered, weighing risks of thrombosis in SCD with risks of bleeding, and we recommend testing for acquired VWD in all patients with platelet count >1 000 000/µL.

Extreme thrombocytosis in a pediatric patient warrants special diagnostic evaluation, including testing for acquired VWD and VWF:GPIbM activity to assess for possible bleeding risk, radiographic evaluation for thrombosis, and evaluation for MPN which includes bone marrow biopsy and genetic testing. Pediatric patients with ET are less likely to have mutations in genes associated with adult ET (*JAK2*, *MPL*, and *CALR*); thus, extended genetic testing should be considered. In this patient, hydroxyurea with aspirin and iron therapy were judiciously used balancing the associated risks of bleeding.

## CONFLICT OF INTEREST

The authors have no pertinent financial disclosures.

## AUTHOR CONTRIBUTIONS

MY: conceptualized the study, contributed to data collection, and wrote the manuscript. GB and SC: contributed to data collection, intellectual process, and manuscript revision. SP: contributed to data and image collection, intellectual process, and manuscript revision.

## ETHICAL APPROVAL

The Institutional Review Board of Children's Healthcare of Atlanta reviewed this report and determined that review and approval by the organization was not required.

## INFORMED CONSENT STATEMENT

Written informed consent was obtained from the patient for publication of this report and accompanying images.

## Data Availability

All data generated or analyzed during this study are included in this published article.
